# The Suppressive Activities of Six Sources of Medicinal Ferns Known as Gusuibu on Heat-Labile Enterotoxin-Induced Diarrhea

**DOI:** 10.3390/molecules19022114

**Published:** 2014-02-18

**Authors:** Hung-Chi Chang, Jaw-Chyun Chen, Jiun-Long Yang, Hsin-Sheng Tsay, Chien-Yun Hsiang, Tin-Yun Ho

**Affiliations:** 1 Department of Golden-Ager Industry Management, Chaoyang University of Technology, Taichung 41349, Taiwan; 2 Department of Medicinal Botany and Health Applications, Da-Yeh University, Changhua 51591, Taiwan; 3 Department of Biological Science and Technology, China Medical University, Taichung 40402, Taiwan; 4 Graduate Institute of Biochemical Sciences and Technology, Chaoyang University of Technology, Taichung 41349, Taiwan; 5 Department of Microbiology, China Medical University, Taichung 40402, Taiwan; 6 Graduate Institute of Chinese Medicine, China Medical University, Taichung 40402, Taiwan

**Keywords:** folk medicine, Gusuibu, heat-labile enterotoxin

## Abstract

Diarrheal disease is one of the most important worldwide health problems. Enterotoxigenic *Escherichia coli* (ETEC) is the most frequently isolated enteropathogen in diarrheal diseases. In developing countries, a very large number of people, especially children, suffer from diarrhea. To combat this problem, World Health Organization has constituted the Diarrhea Diseases Control Program which guides studies on traditional medicinal practices and preventive measures. Gusuibu, a traditional folk medicine, has been claimed to heal certain types of diarrhea. However, so far no scientific study has been carried out on the anti-diarrheal mechanism of Gusiubu. The present study was performed to examine the suppressive activities of ethanol extracts of six sources of folk medicinal ferns used as Gusuibu on heat-labile enterotoxin (LT)-induced diarrhea. Inhibitory effects of six sources were evaluated on the ETEC LT subunit B (LTB) and monosialotetrahexosylganglioside (G_MI_) interaction by G_M1_-enzyme linked immunosorbent assay and patent mouse gut assay. Our results indicated that *Drynaria fortunei* had no anti-diarrheal effect, while, among the remaining five folk medicinal ferns, four belonging to family Davalliaceae had significant abilities on both the blocking of LTB and G_M1_ interaction and the inhibition of LT-induced diarrhea. In conclusion, these findings suggested the potential application of Gusuibu as an anti-diarrheal remedy.

## 1. Introduction

Diarrhea diseases constitute one of the most important worldwide health problems, particularly in developing countries with an estimated 5 billion cases and a mortality of 5 million cases per year. Children less than 5 years of age have been reported to experience higher incidence and severity of gastrointestinal symptoms [1]. Enterotoxigenic *Escherichia coli* (ETEC) are the most frequently isolated enteropathogen, which is responsible for approximately 380,000 deaths annually [2]. Pneumonia and diarrhoea together account for 29% of all child deaths globally. To see a drop in deaths from diarrhea to less than 1 in 1,000, the World Health Organization has constituted the Integrated Global Action Plan for the Prevention and Control of Pneumonia and Diarrhoea which includes studies on traditional medicinal practices together with the evaluation of health education and prevention approaches [3,4,5,6].

Among the vast array of remedies used in the Traditional Chinese Medicine system, many ferns (rhizomes and whole plants mostly) have been recorded to have activities against diarrhea and thus act as very useful remedies [7]. Gusuibu, one such Traditional Chinese Medicine, has a long history of use in the treatment of bone injuries, inflammation, hyperlipemia arteriosclerosis [8]. This folk medicine has also been claimed to heal certain types of diarrhea [9]. However, so far no scientific study has been carried out on anti-diarrheal mechanism or changes the gastrointestinal transit/motility effects of Gusiubu. In the Chinese *materia medica* literature, several species of ferns have been mentioned as Gusuibu [9]. Only a few researches study the chemical composition of the various Gusiubu. *Drynaria fortunei* (DF) contains several flavonones, such as naringin, which present oestrogen-like protective effects in bone [10]. Davallic acid was extracted from *Davallia divaricata* (DD), and its effects on apoptosis induction in A549 lung cancer cells were reported [11]. Some of the proanthocyanidins from *D. mariesii* (DM) could inhibit protein kinase C [12]. 4-*O*-β-d-Gluco-pyranosyl-2,6,4′-trihydroxybenzophenone, which has been isolated from *D. solida* (DS), could bind to the purified C-terminal cytosolic domain of *p*-glycoprotein [13]. As a part of our continuing evaluation of anti-diarrheal activities of Traditional Chinese Medicine materials, the present study was performed to examine the suppressive activities of ethanol extracts of six sources of folk medicinal ferns (*Drynaria fortunei* (DF)., *Pseudodrynaria coronans* (PC), *Davallia divaricata* (DD), *D. mariesii* (DM), *D. solida* (DS), and *Humata griffithiana* (HG)) [14,15] used as Gusuibu on heat-labile enterotoxin (LT)-induced diarrhea.

## 2. Results and Discussion

**Figure 1:**
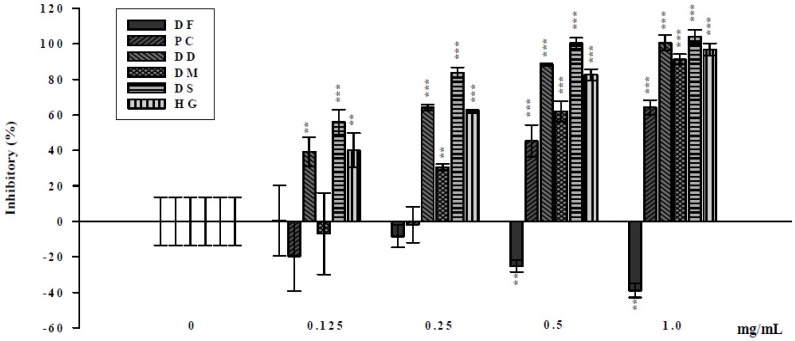
Inhibitory ability of six sources of Gusuibu on the binding of LTB to G_M1_ by competitive G_M1_-ELISA. Different quantities of six sources of Gusuibu were incubated with 16 ng biotinylated LTB at 4 °C for 3 h. The wells were coated with 200 ng of G_M1_ and challenged with 100 μL of biotinylated LTB/compound mixture. Following three washes, peroxidase-conjugated avidin and chromatic substrate were sequentially added. The absorbance was read at 405 nm in an ELISA plate reader. The results are expressed as inhibitory ability (%). Values represent mean ± standard error of four independent assays. ** *p* < 0.01, *** *p* < 0.001, compared with LTB.

The LT is the major virulent factor of ETEC [16] and is a kind of AB toxin, which comprise one A subunit and five identical B subunits [17]. The mechanism of diarrhea induced by LT is initiated by the binding of B subunit (LTB) to the receptor, ganglioside G_M1_, on the surface of intestinal epithelial cells [18,19]. Upon evaluation of the inhibitory ability of the six sources of Gusuibu on the binding of LTB to G_M1_ by competitive G_M1_-ELISA it was observed that five ferns (PC, DD, DM, DS and HG) blocked the binding of LTB to G_M1_, resulting in the suppression of LT-induced diarrhea. All four species belonging to family Davalliaceae were found to be more efficient compared to two species of Polypodiaceae (Figure 1). All Davalliaceae species were significantly more efficient than the Polypodiaceae species at 0.25 mg/mL.

**Figure 2:**
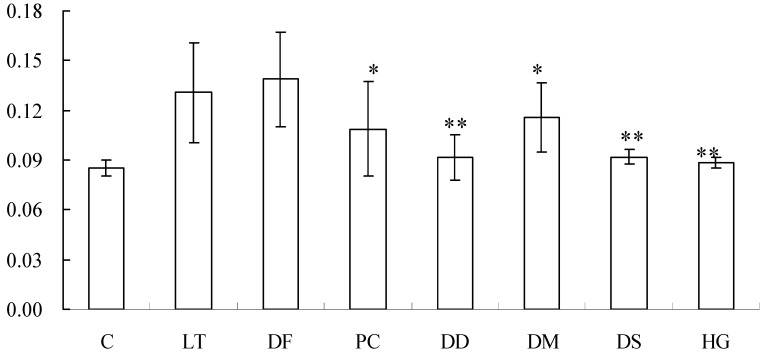
Anti-diarrheal effect of six sources of Gusuibu by patent mouse gut assay. Three mice per group were administrated orally with PBS, 10 μg LT, or in conjunction with different sources of Gusuibu. After 6 h incubation, mice were sacrificed. The gut/carcass weight ratio was calculated for each group. Values represent mean ± standard error of triplicate assays. C: control. * *p* < 0.05, ** *p* < 0.01 compared with LT.

Since one of the biological activities of LT is the induction of fluid accumulation in the intestine, we analyzed the anti-diarrheal effect of the six ferns by a patent mouse gut assay. As shown in Figure 2, LT stimulated the fluid accumulation in the gut, with the mean gut/carcass weight ratio as 0.13. Five ferns (PC, DD, DM, DS and HG) suppressed LT-induced fluid accumulation. Out of these five sources, four (DD, DM, DS and HG) belong to the same family, while three (DD, DM and DS) are the same species.

Thus, our results indicate that DF had no anti-diarrheal effect, while, among the remaining five species, four belonging to family Davalliaceae had significant ability to inhibit LT-induced diarrhea indicating their potential application in the anti-diarrheal remedies.

## 3. Experimental

### 3.1. Plant Materials

Rhizomes of *Drynaria fortunei*, *Pseudodrynaria coronans* (PC) (both from the Polypodiaceae), *Davallia divaricata*, *D. mariesii*, *D. solida*, and *Humata griffithiana* (HG) (all four from the Davalliaceae) used as Gusuibu were collected from the counties of Hsinchu, Taichung, Nantou and Taitung in Taiwan (Table 1). These were identified and authenticated by Professor C.C. Chen of the Institute of Chinese Pharmaceutical Science, China Medical University, Taichung, Taiwan.

**Table 1:** Particulars of the six sources of medicinal fern used as Gusuibu, and their comparative yields in ethanol extracts.

Family Name	Botanic Name	Common Name	Voucher Number	Location	Yield (%)
Polypodiaceae	*Drynaria fortunei* (Kze.) J.Sm.	Gusuibu	CMU-94-DF-01	Hsinchu, Taichung	11.2
	*Pseudodrynaria coronans* (Wall. ex Mett.) Ching	Gusuibu	CMU-94-PC-01	Taichung, Nantou	15.0
Davalliaceae	*Davallia divaricata* Bl.	Dayegusuibu	CMU-94-DD-01	Nantou	14.6
	*Davallia mariesii* Moore ex Bak	Haizhougusuibu	CMU-94-DM-01	Nantou	8.5
	*Davallia solida* (Forst.) Sw.	Koyegusuibu	CMU-94-DS-01	Taitung	12.7
	*Humata griffithiana* (Hk.) C.Chr.	Begaigusuibu	CMU-94-HG-01	Taichung	6.7

### 3.2. Extractions

Dried rhizome (100 g each) was macerated with 95% ethanol (1,000 mL) for 24 h at room temperature. Filtration and collection of the extract was done three times. Then, the ethanol crude extract (~3,000 mL) was evaporated to 10 mL and then dried *in vacuo* at 40 °C (Table 1). The dry extract was weighted and stored in −20 °C until further use.

### 3.3. The *In Vitro* and *In Vivo* Assay

The methods of (i) Expression and purification of *Escherichia coli* LT and LTB; (ii) Patent mouse gut assay; (iii) Biotinylation of LTB and (iv) Competitive G_M1_-ELISA were followed as per our previous report [14,15]. Mouse experiments were conducted under ethics approval from China Medical University Animal Care and Use Committee.

### 3.4. Statistical Analysis

Data were presented as mean ± standard error. Student’s t-test was used for comparisons between two experiments. A value of *p* < 0.05 was considered statistically significant.

## 4. Conclusions

We demonstrated that the ethanol extracts of six sources of folk medicinal ferns used as Gusuibu suppressed LT-induced fluid accumulation through blocking the binding of LTB to G_M1_. *Drynaria fortunei* had no anti-diarrheal effect, while other five ferns had significant abilities on both the blocking of LTB and G_M1_ interaction and the inhibition of LT-induced diarrhea. Therefore, these data suggested that *Pseudodrynaria coronans*, *Davallia divaricata*, *D. mariesii*, *D. solida*, and *Humata griffithiana* might be candidates for the treatment of LT-induced diarrhea.
